# Smart Structural Monitoring: Real-Time Bridge Response Using Digital Twins and Inverse Analysis

**DOI:** 10.3390/s25113513

**Published:** 2025-06-02

**Authors:** Sanduni Jayasinghe, Zhiyan Sun, Amir Sidiq, Mojtaba Mahmoodian, Farham Shahrivar, Sujeeva Setunge

**Affiliations:** School of Engineering, RMIT University, 124 La Trobe Street, Melbourne, VIC 3000, Australia; s3751824@student.rmit.edu.au (S.J.); s3950682@student.rmit.edu.au (Z.S.); amir.sidiq@rmit.edu.au (A.S.); s3737042@student.rmit.edu.au (F.S.); sujeeva.setunge@rmit.edu.au (S.S.)

**Keywords:** structural health monitoring, structural integrity assessment, digital twin, finite element method, inverse method

## Abstract

Continuous monitoring is significant to ensure the safe operation of infrastructure systems despite the high costs of traditional methods. The current study presents the development of a real-time digital twin of a laboratory-scaled bridge that can assist in the infrastructure monitoring process. Initially, the bridge model was instrumented with strain gauges, and a script was developed to conduct an inverse structural analysis and subsequently, run a finite element analysis to visualize the overall structural response. Three main loading scenarios were tested, and observations highlighted that the digital twin model emulated the actual structural behavior with a high accuracy. Also, the magnitude and the location of the applied loads on the real structure were correctly identified and a linear elastic behavior was identified in the digital model as expected from the actual structure. Further, the rates of change in the strain values and deflections were also evaluated while discussing the significance of digital twin development.

## 1. Introduction

A reliable transport infrastructure depends heavily on a modern, well-maintained road network. Such a road network significantly depends on the functionality and serviceability of bridges, as they play a crucial role in the interconnectivity of transportation systems. On the other hand, bridges experience mechanical and environmental impacts that cause structural deterioration during their operational life, therefore leading to structural failures [1]. For instance, the effects of adverse weather conditions, fatigue damage, material depreciation and corrosion damage are inevitable in an aging bridge. Consequently, bridges will be gradually degraded while losing their performances in both functional and structural aspects with age [2,3]. According to recent reports, in Australia, there are more than 53,000 bridges, whereas approximately 55% of highway bridges have passed approximately 20 years of their design life [4]. In the United States, there are approximately 600,000 bridges and more than 42% of these bridges have surpassed nearly 50 years of their design life, whilst approximately 7.5% of bridges have been categorized as structurally deficient [5]. Therefore, there is a need for attention to be paid to aging bridges as unsatisfactory monitoring and maintenance practices can potentially lead to sudden and catastrophic failures. Statistical data show that more than 60 bridges have collapsed worldwide during the past decade due to low and/or inappropriate maintenance. For example, the Morandi Bridge in Genova, Italy, collapsed in 2018 and was known as a tragic failure that resulted in 43 lost lives and many injuries [6]. Recently, another bridge collapsed (suspension bridge type) in Gujarat, India, in 2022 and caused the loss of more than 134 lives and many injuries [7]. Therefore, it can be noticed that the consequences of bridge failures are fatal and irreversible and there is a significant need for proper and reliable bridge management practices to ensure the health and safety of citizens. Such bridge management and structural health monitoring systems would have significant impacts on the social and economic security of a country [8].

Under the current bridge asset inspection and maintenance procedures, inspectors are generally required to attend the bridge site, visually anticipate the severity of the bridge conditions and report the conditions, then the appropriate maintenance practice will be considered by expert judgment. The bridge owners would then implement and update their official databases [2]. This process can be complex, depending on the number of bridges within the infrastructure system of the country, and a high number of workforces is required to conduct such inspection procedures. In Australia, more than 800 organizations are assigned to bridge management and maintenance activities [4]. The bridge management databases should also be updated following maintenance activities [9]. Furthermore, non-destructive testing (NDT) techniques can also be applied to obtain more detailed visual inspections under the SHM process. Those techniques will be really useful in identifying the magnitude and location of damages. However, the accuracy of NDT techniques is highly dependent on the sensitivity of the equipment and a trained workforce, whilst the cost of equipment is also at a higher rate [10]. Accordingly, regular inspections are more time-consuming and are associated with high costs. Statistical data shows that, in Australia, approximately $19 billion is annually spent for maintenance activities for the road network and it has been identified that the aging bridges are the major reason for this extensive expenditure [11]. Studies have found that the cost of the annual maintenance of bridges ranges from 0.4% to 2% of its construction cost. For instance, for a bridge having a designed life of 100 years, the cost of maintenance would be doubled compared to the initial cost of construction [12]. Similarly, it has been reported that, in most of the developed countries, the associated costs of maintenance are increasing drastically due to inappropriate bridge maintenance and rehabilitation procedures [5,13]. Therefore, there is a need for a more intelligent approach to monitor and maintain bridge systems remotely over the traditional on-site bridge inspection and/or NDT testing procedures. It is rare to see such attempts and paradigms including the real-time monitoring of bridge behavior when experiencing the mechanical and surrounding environmental conditions. The use of the Digital Twin (DT) has the potential to allow for intelligent and reliable bridge health monitoring and maintenance.

The twin concept was first utilized by NASA in 1969 to oversee the performance of an aircraft when training the flight crew during the operation of the Apollo program [14]. In 2002, Michael Grieve proposed the concept of a digital twin in their product life-cycle management architecture [15]. In 2012, NASA launched an official announcement in relation to the development phases of a DT by introducing novel methodologies for DT implementation [16]. Since then, utilization of a DT has captured constant attention from academics and industrials [17], and it is becoming a critical component of Industry 4.0 [18]. In the civil engineering discipline, DTs are deemed to be the live digital depiction of an existing structure, or system, or system of systems (i.e., a network) to advance their functionality through proper monitoring and control measures [19,20]. It is apparent that DTs incorporate data and services as significant counterparts, in addition to the physical structure, digital model, and the communication channels between them, in order to facilitate bridge health monitoring (BHM) practices. Currently, there is a significant demand worldwide for bridge health monitoring systems aided with real-time sensing since this method assists in ensuring the safe operation of bridges. Such a system would require sensing and recreating the deformed shape of a bridge intelligently incorporating real-time measurements [21]. The implementation of DT in the field of BHM can be identified in several major steps: (1) data acquisition from an operating asset, (2) development of the virtual model, (3) connectivity between the operating asset and virtual model and (4) analysis and evaluation of the structural health assessments based on the collected data [22,23,24]. Consequently, BHM has the potential to perceive critical failure modes of structures while determining any undesired stress concentrations, deformations and extrinsic loads [25]. Generally, bridges would be monitored either by gathering response-based (strain, tilt and displacement), geometry-based (scanning and surveying), vision-based (video or image) or loading-based (weather and operating conditions) [26,27] data.

Bridge digital twins can be identified as living replicas of physical bridges and are able to visualize all relevant aspects of target bridges, including their geometries, material properties, site-specific boundary conditions structural responses, etc. This requires the physical bridges to facilitate real-time data transfer by common data frameworks that are accessible to all stakeholders [28]. Spontaneously, as the sensor network measures the structural response and transfers it to the digital model, it should be able to determine the structural responses virtually. This continuous link between the physical and virtual models is typically maintained through data acquisition systems integrated with edge or cloud computing platforms. Additionally, tools such as embedded sensors, data loggers and analytical engines also play a crucial role in ensuring seamless communication, rapid data interpretation and accurate virtual replication of the physical structure [29,30]. However, this is challenging for bridge owners attempting to anticipate any structural breakdowns and trigger warnings by comparing calculated responses to measured readings within a short time frame [16]. There are different vendors, such as Autodesk, Bentley, ClearEdge3D and AVEVA, that provide commercial software packages for developing digital twin environments, primarily for design visualization and asset information management. Platforms such as Bentley’s iTwin and Microsoft Azure Digital Twins have also seen growing adoption in infrastructure and building management as they offer capabilities like IoT integration, 3D modeling and real-time status monitoring. However, one of the drawbacks of the commercial software packages is that the vast majority of the platforms only accommodate geometric digital twins for visualizing purposes at the design stage [9]. Some of them are data-driven at the operational layer and often lack embedded physical modeling capabilities. Accordingly, they are limited in their ability to simulate structural behavior under a load, predict stress or deformation fields, or support detailed structural health assessments. From these limitations arises the demand for having physics-based digital twins that are capable of integrating real-time sensor data with finite element (FE) modeling to provide insights into structural performance. Unlike the geometric digital twins, physics-based twins always enable the evaluation of internal stress distributions, displacement patterns and stability conditions, while paving the path to form a more comprehensive approach to real-time monitoring and intelligent maintenance for civil infrastructure.

Moreover, digital modeling and health assessments of a physical asset can be implemented as a physics-based, model-driven method or as a data-driven, measurement-based method [31]. The physics-based approach utilizes fundamental physical laws in order to identify the underlying structural behavior while the other approach is a data-driven approach that uses the trends, correlations and patterns of a statistical model to determine structural behavior. A physics-based approach is considered to be superior to a purely data-driven approach, as it is more comprehensive in considering the governing factors of the structural system, while a data-driven approach is more statistical in nature and would help in real-time simulations [28], allowing users to quickly process the data in order to get a quick output [32,33].

Digital Twins (DTs) can be a versatile BHM tool, offering functions such as data storage, performance assessment and fault diagnosis of bridge assets [34,35,36] Although researchers have used both physics-based and data-driven model development techniques to create virtual models of bridges, most of the studies have mainly considered the digital visualizations of bridges that include an FE model representation [28,37,38,39,40] rather than a real digital twin, which continuously receives data from sensors installed on the structure. Amongst the physics-based model development techniques, FE modeling is a more promising approach for this purpose [38,41,42]. However, due to the inherent limitations of the conventional FE method from its high computational cost (i.e., both computational power and time), real-time analysis is indeed challenging even for super computers [43]. Also, as the measured structural response will be the input for the physics-based approach, an inverse method of structural analysis or a shape-sensing technique is required to simulate the structural health of the physical counterpart [41]. Nevertheless, such a system is highly likely to be ill-conditioned due to either unavoidable or minor errors from the measuring devices, or insufficient or suboptimal sensor placement. For instance, a small deviation in a strain gauge reading can lead to a disproportionately large error in the estimated loads [44,45,46]. Further, Stevens [47] and Okubo et al. [48] emphasized that such instability arises due to dominant terms in the system matrices and sensitivity to noise. Hansen and Starkey [49] also showed that it is important to investigate the optimal sensor placement in the structure as it influences the condition number of the inverse system, and accordingly, it assists in overcoming the ill-conditioning issue of such systems. Consequently, that should lead to investigating a more stable, accurate and efficient method to determine the behavior of a structure in real-time.

In this study, a novel physics-based technique that captures and visualizes the structural behavior under real-time conditions has been developed. Such a novel technique has been implemented for a digital twin creation for a truss bridge at a laboratory scale. The proposed digital twin system used the novel method that uses integrated inverse load identification and the finite element method approach in real time. The system captures real-time structural behavior via the strain variations within the structural members of the bridge while evaluating the stress distribution and deformations by back-calculating the applied load. In addition, this system allows us to visualize the real-time structural behavior of the bridge in a user-friendly manner. The results have been used to identify and evaluate the structural integrity of the bridge model. The study can be used in real-world, large-scale applications and allows the asset owners to easily identify the locations of possible failures to prioritize maintenance activities for the vulnerable areas of the bridge. The potential integration of data-driven components remains a promising direction for future research, particularly for reducing sensor dependency and improving robustness.

## 2. Inverse Load Identification Method

A numerical model is supposed to be developed in a virtual environment while avoiding the actual physical context in FEM simulations [50]. Additionally, it is required to deploy accurate loading and boundary conditions. Hence, with the identified principles of dynamics and elasticity, the structural response of a structure can be simply obtained when the time-varying loads are known [44]. Such a type of situation is therefore known as a forward problem. It is possible to measure the loads on real structures using load cells; however, due to the inconsistency of the loading patterns acting on a real structure, such load cells have a limited capacity in generating an overall structural response [44]. For example, Dan et al. [16] developed a digital twin system using WIM (Weigh-in-motion) and multi-source heterogeneous machine vision techniques to measure the vehicle loads acting on a group of regional bridges, which are situated in Shanghai, China. Whilst the bridge can be subjected to different types of loading patterns, the authors have considered only the traffic load here to make it into a forward problem, and they highlighted the traffic loads as the most straightforward method to connect the physical bridge and virtual bridge in a digital twin system. Despite that, another drawback was the possibility that the load measurement obtained may be incorrect due to different factors such as the manual data transferring, coordinate transformations and offline data acquisitions, which potentially caused significant errors in visualizing and analyzing the structural integrity assessment.

Moreover, in BHM procedures, generally, the structural response is measured, and such measurements will then be used to determine the condition of that structure and hence, it will be an inverse approach. Also, it can be noticed that the inverse approach cannot be achieved directly, therefore an inverse method is highly ill-conditioned, which in most cases misleads the load approximations. In fact, the estimated loads could be largely deviated even from a small variation in the measured response [44]. Also, there might be alternative loading patterns that can induce the same structural response and accordingly, the load estimations may be erroneous. The current study has implemented a novel technique that will allow the user to digitally capture and visualize the structural behavior under real-time conditions. Such a technique is an integration of the inverse load identification method that is explained here and the finite element method.

Additionally, one of the concepts that was found in the literature is the inverse load identification method, which was first proposed by Masroor and Zachary [51]. This inverse load identification method is applicable only under several circumstances; thus, the structure should behave linearly and the proportionality between the load and strain could be assumed in the event of interest [52]. If such conditions are satisfied, then the following expression (Equation (1)) can be presented in between the load and the strain values [44].

(1) $$\left\{\varepsilon \right\}=\left[A\right]\left\{f\right\}$$

where $\left\{\varepsilon \right\}$ is $\left(g\times 1\right)$ matrix, g is the location of strain gauges, $\left[A\right]$ is $\left(g\times n\right)$ matrix, which is known as the sensitivity matrix, and an element of this matrix $\left(a_{ij}\right)$ will be the strain at ith location due to the applied unit load at location j. $\left\{f\right\}$ is $\left(n\times 1\right)$ matrix, which shows the applied loads on the structure. Then, the loads that are acting on a structure can be obtained as in the following expression (Equation (2)).

(2) $$\left\{f\right\}={\left({\left[A\right]}^{T}\left[A\right]\right)}^{-1}{\left[A\right]}^{T}\left\{\varepsilon \right\}$$

The sensitivity of the matrix $\left[A\right]$ is ${\left[A\right]}^{T}\left[A\right]$. Therefore, for accurate estimations of loads, minimization of the sensitivity will be required. Moi et al. [41] followed this method to implement a digital twin for a knuckle boom crane that includes a moving mechanism, and the cable tension of the crane was accurately identified in real-time. However, equal numbers of strain gauges and applied loads were required for their case and only the cable tension was obtained in real-time rather than showing the whole structural response. Nevertheless, the existence of sensors at all the elements of a truss structure or a frame structure is required for this method to obtain the most accurate results. Despite that, such work is only for structures with shell/plate elements and is mostly applicable for linear events.

In real-world applications, the sensitivity matrix $\left[A\right]$ is also prone to errors arising from several sources, including sensor misplacement, manufacturing tolerances, material variability and environmental effects. These inaccuracies can lead to ill-conditioning, where small errors in the strain reading produce large errors in the estimated loads. Such sensitivity is a known limitation of inverse load identification methods. Gupta [44] has introduced three different methods to minimize the sensitivity, which are as follows: (1) optimizing the strain gauge locations, (2) the number of gauges and (3) the angular orientations. This process has been utilized for dynamic load cases by Gupta [44] and the $\left\{f\right\}$ was the dynamic loads or mode shapes for dynamic instances. Additionally, previous researchers have suggested Tikhonov regularization and singular value filtering as well to control the condition number of the system and mitigate the effects of noise and calibration errors [2,44,45,46]. In the current study, these effects were minimized by using a controlled laboratory setting, accurate strain gauge installation and validated model geometry. However, future work should address the stability of this approach under more realistic, uncertain conditions, and incorporating statistical filtering techniques and adaptive calibration strategies will be essential steps for deploying this method in field-scale digital twin applications.

## 3. Experimental Program

In this study, a 2D truss structure is considered to represent the main structural component that carries the loads from a 3D truss bridge. In a typical 3D truss bridge configuration, each side truss primarily resists loads acting within its own plane. The deck system manages the transverse distribution of loads by transferring vertical and lateral forces between the two side trusses through horizontal deck beams. As a result of that, the in-plane axial behavior of each 3D truss configuration can be effectively studied using a 2D representation. Therefore, the evaluation of the inverse load identification technique and real-time structural response monitoring is easily achievable for a 3D truss bridge with this simplification.

### 3.1. Equipment and Devices

**Figure 1:**
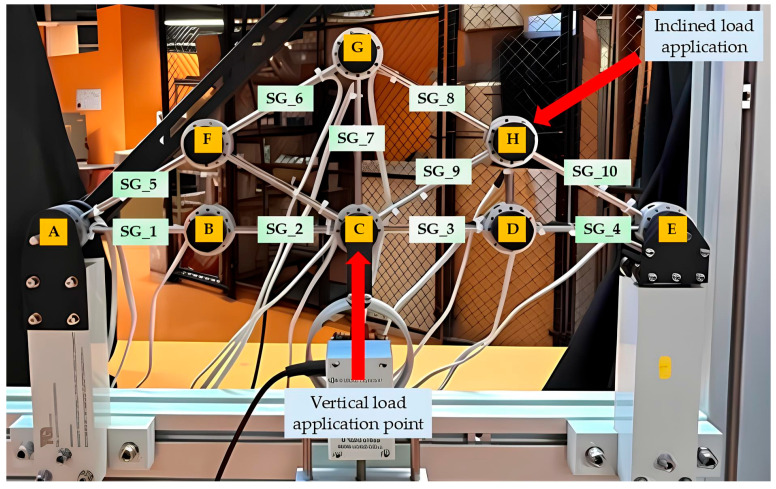
Detailed experimental setup.

The STR8 pin-jointed symmetrical truss bridge model available at RMIT University, Melbourne, Australia was used as the experimental structure in the current work. The symmetrical truss contains 13 members in circular cross sections of diametrical size of 6 mm. These members are made from steel with a Young’s modulus of approximately 193 GPa (Figure 1). The truss structure is supported by using a pin support at one end and a roller support at the other end. This truss structure has a maximum capacity of applied loading of compression and tension ranging from −500 N to +500 N, respectively. The loadings can be applied at any pin-joints and any angles up to 45°, which are measured and displayed by embedded load cells within the loading mechanism.

**Figure 2:**
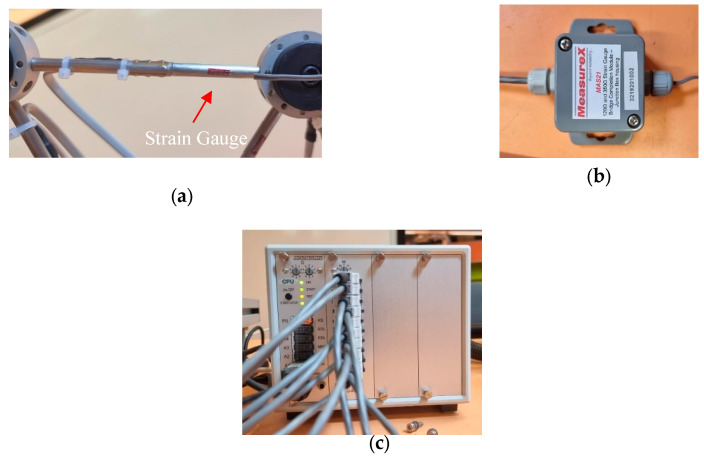
(**a**) Strain gauge attached to one member. (**b**) Junction box. (**c**) Data logger.

Figure 1 shows the experimental setup that was used for the testing. A new set of gauges that includes ten strain gauges manufactured by Measuring Instruments Laboratory Co., Ltd., Tokyo, Japan was used for the current study. The strain gauges adopted in this research are 120 Ohm quarter bridge with two-wire sensors and attached onto the experimental truss structure with an adhesive product as shown in Figure 2a. It can be noticed that there is an existing wire loom on the truss structure due to the manufacturer-installed sensors on several members; however, for this study, strain gauges are required for almost every member to deploy the inverse load identification. All strain gauges (SG_1 to SG_10) were installed at the midpoints of each truss member, as illustrated in Figure 1. Additionally, node A (on the left side of the truss model) represents a roller support, while node E (on the right side) serves as a pin support.

The strain data obtained were then transferred to a bridge completion module MAS21 by MeasureX that supports the completion of these quarter bridge sensors, Figure 2b. Then, the data were transferred to a data logger box, which was obtained from Almemo measuring devices, Figure 2c. The data acquisition logger converts electric resistance measurements from the strain gauges and converts them into numerical digits. This information is then transferred into the developed Python code for structural analysis in a real-time manner. The data logger was connected to the computer by a USB cable for a better connection. However, it comes with the facility to connect with the computer remotely.

### 3.2. Sensor Locations

**Figure 3:**
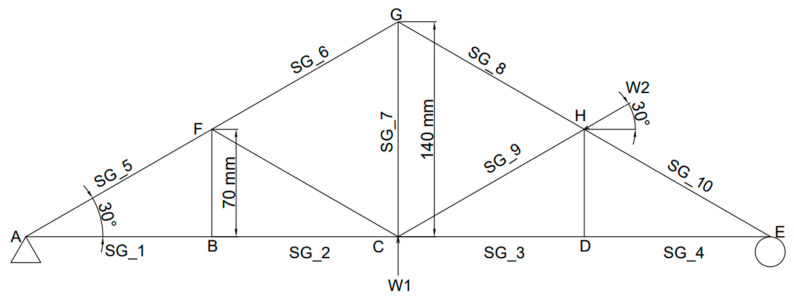
Geometry, structural setup and sensor configuration of laboratory truss model.

The optimum number of sensors and locations have been deployed considering the zero force members in the truss. This led us to select ten members from the entire truss structure (i.e., out of 13 members) to be instrumented with a strain gauge and directly connected to the data acquisition module. For the other three uninstalled truss members, the strain responses are assumed to be faulty, and thus negligible with zero readings. This includes two vertical and an inclined member. Figure 3 shows the schematic location of the strain gauges (SG) and loading cells (P). The locations of loading cells were also selected in such a way that those uninstrumented members have zero stresses and those two locations were fixed throughout the experimental life.

### 3.3. DT Development

**Figure 4:**
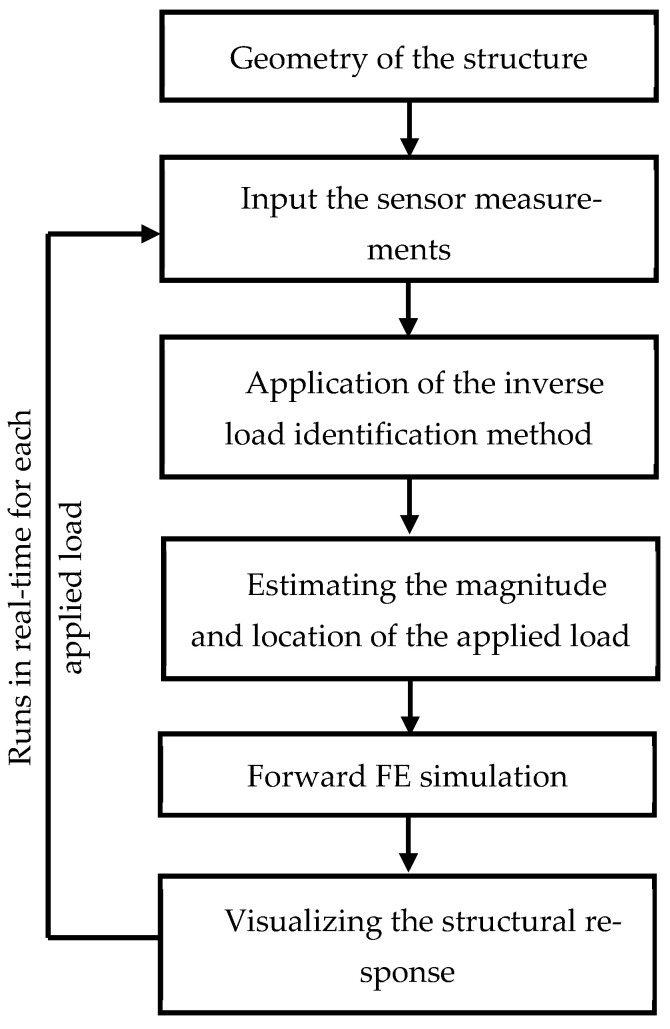
The developed methodology for the creation of the digital twin of the truss bridge.

Following the previously described major steps, sensors and data logging devices were employed as telemetry data collection units that gather the dynamic structural responses of interested bridge structure. The strain measurements from the strain gauges are continuously transmitted to the virtual model, which processes them using an inverse load identification algorithm developed in Python and illustrates virtual structural behaviors. Figure 4 illustrates the systematic steps undertaken during the digital twin development.

When a load is applied to the physical structure, it will be deformed and will alter the internal force distribution, and that will be captured by the strain gauges. They will record a strain measurement at that time, and it will be transferred to the virtual model via the data logger. This virtual model employs a sensitivity matrix, which has been predefined based on the structural characteristics to calculate the magnitude and the location of the applied loads. This inverse estimation method has been outlined in Section 2. Once the loads are calculated, the virtual model executes a forward finite element analysis in Python to compute the resulting stress and displacement fields across the entire structure. Simply, this is a two-step process that conducts real-time inverse load identification followed by an FE simulation. Ultimately, the actual structural behavior of the physical structure will be reflected in the virtual model. The updated structural states are stored and visualized for monitoring or further analysis.

The continuous link between the physical system and the digital model ensures that structural responses are tracked in real-time while creating a closed-loop system for monitoring and simulation. This framework forms the computational foundation for the real-time structural assessment capabilities of the digital twin.

### 3.4. Validation

In order to obtain the maximum accuracy of the installed strain gauges and the basis for the digital twin model validation, a calibration process is required. Initially, the applied loading cells were set to zero within the experimental apparatus, which shows zero stress value in the members at this instance, as measured by strain gauges. Then, compression and tension loads were applied to the structure gradually in negative and positive directions, respectively. Each time the load returns to zero, the stress and deformation measures are checked for returning to the original reference point. As it was returned to calibrated reference points, the model was deemed to be validated.

**Figure 5:**
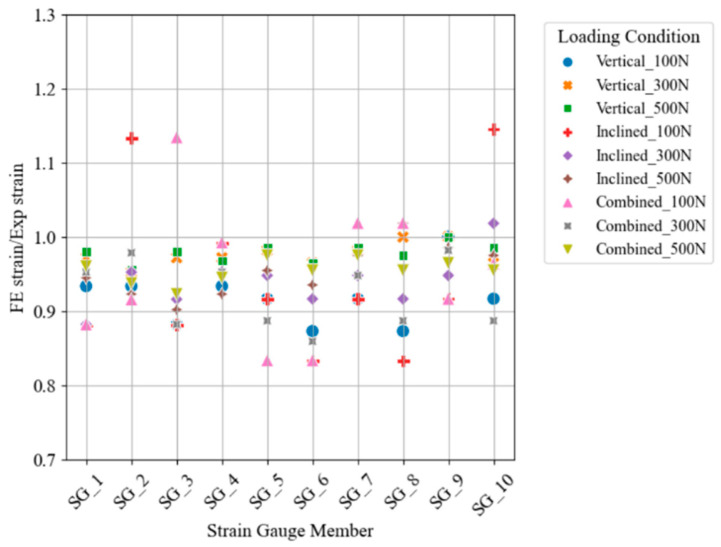
Validated structure of virtual model when loads were set to zero.

The loads were applied to the structure from 0 N to 500 N in 100 N intervals with reference to the readings of the load transducers, and the loads and stress values were estimated by using the developed Python script. The location of the load was accurately identified by detecting high magnitude estimated loads in the locations where the loads were applied, with a maximum deviation of less than 15% compared to the experimental load cell readings. This deviation reflects the maximum absolute error between the predicted and measured loads across the range of testing values. Figure 5 shows a scatter plot of the strain ratio between the numerical and experimental values, indicating that all the ratios fall within the range of 0.85 to 1.15. The inclined load application was identified as a horizontal and vertical component at the loading point and the result was similar to the load application. Therefore, the accuracy of the developed model has been identified to be at a high enough level to continue the development of the digital twin of the actual bridge.

## 4. Results and Discussion

In the initial stages of the experiment, we attempted to use the manufacturer-installed strain gauges on individual members of the truss structure and progress the inverse load identification method on the truss bridge. However, it was observed that the inverse load identification method requires typical strain gauges at each element to function properly. Ideally, any target bridges should be excessively populated with multi-directional strain gauges for the most comprehensive structural response collections. Realistically, the number of available sensors are more often limited by financial and practical aspects. In addition, due to vandalism and environmental factors in the field, not all gauges would be functioning throughout the installation period. In order to simulate the laboratory conditions with real applications (i.e., existing structures) and test the tolerance of the adopted load identification method, only 10 out of all 13 truss members were installed with strain gauges. Based on the results, the stresses that each truss member undertakes were visualized in the virtual model together with the deflections. For better visualization of the deformed model, the deflected shapes were scaled by a factor of 250. According to the output of this process, one can determine where this model shows the highest stress and maximum displacements, and by extending this method into a real structure, it is possible to monitor its real-time behavior.

**Figure 6:**
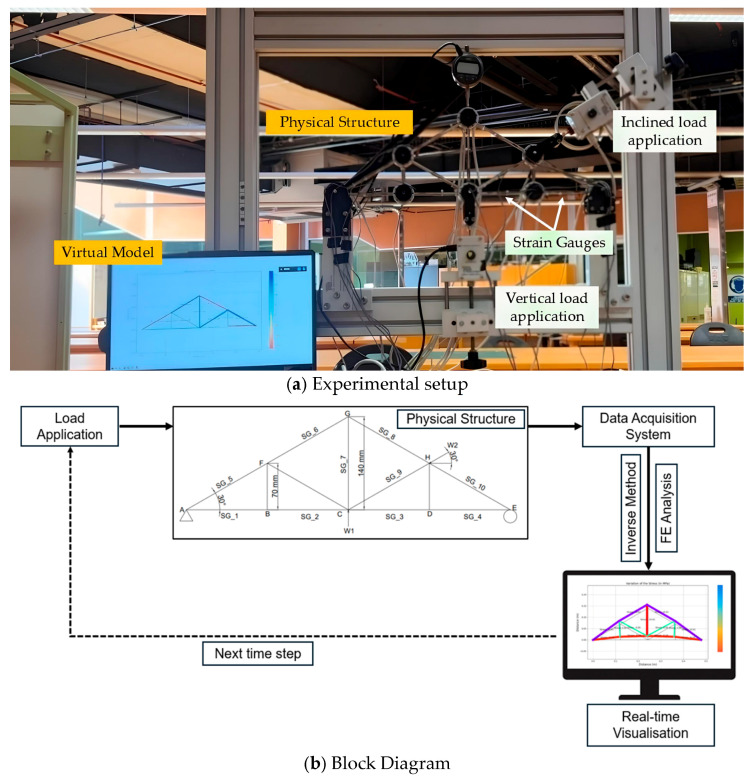
Digital twin of the laboratory truss bridge.

In this experimental study, using a limited number of strain gauges, the digital twin model was designed effectively so that it can visualize the real-time structural behavior under different types of static loads that would potentially act on any location of the actual truss model. Initially, the truss structure was tested for vertical loads, then inclined loads, and later for a combination of both. In all of these loading scenarios, the applied load was at three different levels including 100N, 200N and 300N. First, the vertical load (V) was applied upwards at the middle-bottom node of the truss (node C), as shown in Figure 3 as P1. Then, the inclined load (I) was applied at node H as shown in Figure 3 as P2. Finally, for the combined loading (C) scenario (i.e., vertical and inclined), the load was applied simultaneously at the same rate and conditions. In order to verify the sensitivity of the digital twin model for lower and higher loading ranges, the loads were applied by gradually increasing them within the capability of the truss bridge model, and then the simulation data of the virtual model was evaluated in terms of real-time strain variations and real-time stress variations. To ensure consistency and repeatability, each test was repeated three times. Figure 6 shows the setup of the digital twin model, including both the virtual model and the digital model.

### 4.1. Real-Time Strain Variations

**Figure 7:**
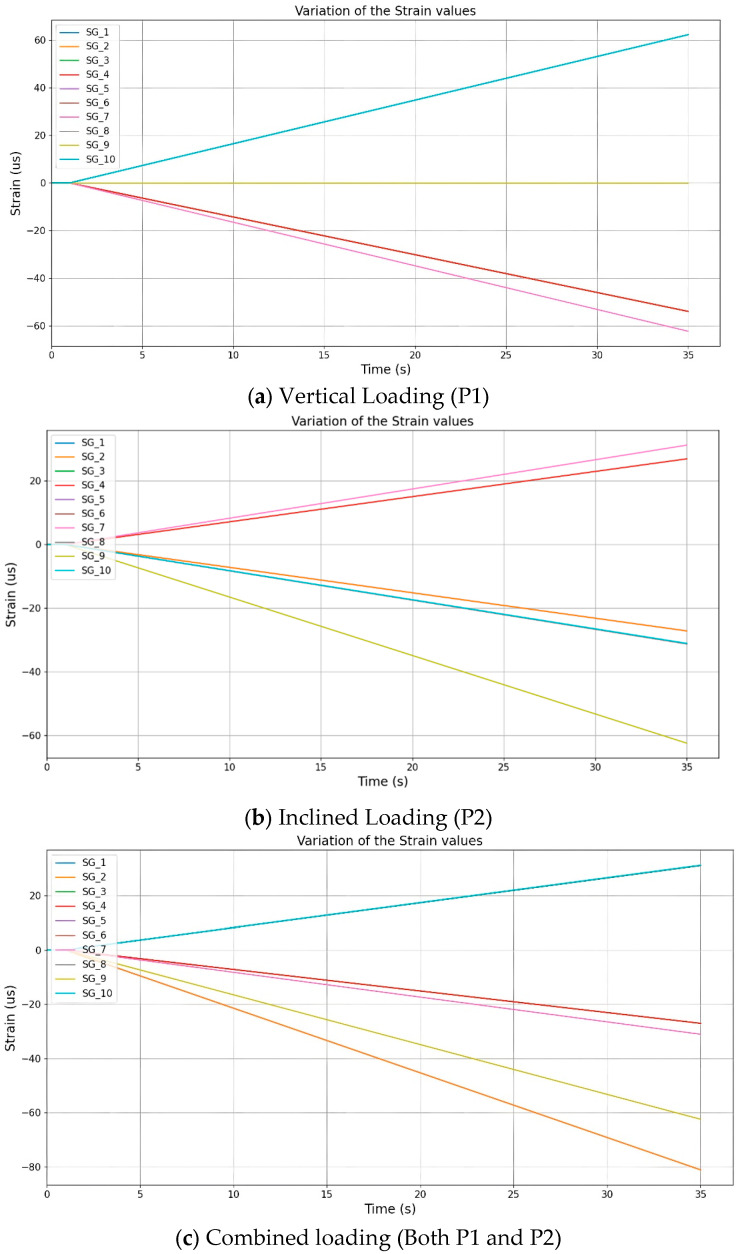
Variations of values of strain gauges with time.

The strain measurements were obtained and monitored in real-time for the three mentioned loading scenarios (i.e., vertical, inclined at 30^o^ and a combination of both) by increasing the load from 0 N to 500 N at a constant rate (10 N/s). Figure 7 shows the strain variation of each member from 0 to 35 s. The strain variations for different loading scenarios were determined by inversely calculating the applied load using strain gauge readings on each member. This calculated load was then applied to the truss structure to confirm the high sensitivity of the developed digital twin. The strain values are shown in micro strain whilst the time is shown in seconds. The vertical load is acting upwards at node C, and the inclined load is acting diagonally (i.e., 30^o^) on the truss model at node H. Also, as the sampling rate of the sensors can be changed as per user requirements, for this experimental program, it was set to 1 s and the refresh rate of the digital model was also set to 1 s to obtain a near real-time response. Figure 7a–c show both positive and negative strain values where the positive strain values indicate tensile stresses and the negative strain values indicate compressive stresses in the members of the tested truss. It can be noticed that the strain variations of all the members showed nearly perfect straight lines. An explanation for such a performance is due to the applied load increasing at a constant rate. The strain values are within the range of the Young’s modulus of the material of the truss structure.

Seemingly, as in Figure 7a, when a vertical load is applied at node C, strain gauges 1, 2, 3 and 4 show the same form of strain variation (i.e., SG member). Similarly, the strain gauges 5, 6, 8 and 10 (i.e., SG member) also show the same form of strain variation. Such a mechanism of occurrence can be due to the symmetry of the truss structure design. Alternatively, the value of strain gauge number 9 is zero for this loading arrangement, and effectively the member that includes SG_9 carries a zero-axial force. Similarly, it was observed that the members connected to nodes BF, CF and DH (Figure 3) carry zero forces for this loading arrangement (i.e., instrumented). Consequently, it was observed that the model had a highly accurate performance in reflecting the strain variation of each of the members under the vertical loading arrangement.

When inclined loading was applied from node H, it was expected that the structure would behave differently in comparison to vertically applied loading (Figure 7b). Thus, the inclined loading arrangement leads the truss structure to behave unsymmetrically, and hence, the strain values vary for most of the members. For instance, SG_1 and SG_2 follow the same form of variation, in comparison to the members SG_3 and SG_4, which also follow the same form of variation. Effectively, the members SG_5, SG_6, SG_8 and SG_10 follow the same form of variation in (Figure 3, with very minor changes in their variations, which are difficult to visualize in the graph, but were was reflected when the numerical values were compared with each other. While the member SG_9 has the highest strain variation in comparison to the remaining members for the inclined loading scenario, it can be noticed that for the same member (SG_9), the strain variation was zero for the vertical loading scenario. It can be determined that the location of the applied loading has a significant effect on the structural behavior for this truss structure. Moreover, for combined loading (i.e., vertical and inclined applied simultaneously), while the load changing rate of the inclined load and the vertical load were kept at the same frequency, the rate of strain for the member is different in comparison to the earlier two loading scenarios (i.e., vertical or inclined). Figure 7c shows the strain variation of each member under such a loading arrangement. It is clear that the strain variations for members SG_1 and SG_2 follow the same form of variation in comparison to SG_3 and SG_4, which also have the same form of variation. However, for members SG_5, SG_6, SG_8 and SG_10, a very similar variation was obtained. All these observations are similar to the inclined loading scenario (i.e., for tension or compression) including that of SG_9. However, the gradients of strain variations are different in comparison to the inclined loading scenario. The combination of the different loading scenarios indicates that adding a symmetrical load to an unsymmetrical loading arrangement has a minimal effect on the structural response while it has an effect on the rate of strain changing.

**Figure 8:**
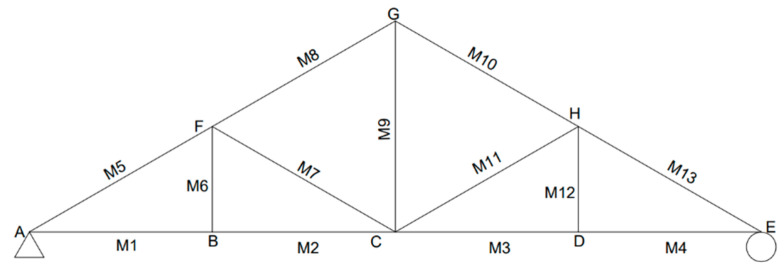
Numbering order of members and node labelling of the truss model.

**Figure 9:**
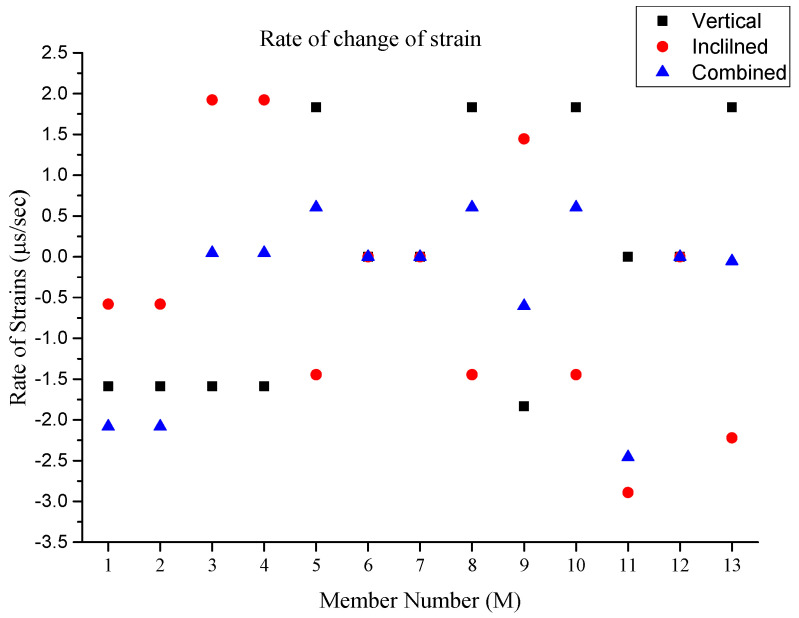
Rate of change in strain of each member in the truss.

In order to further evaluate the effectiveness of the strain rate variations for the different loading scenarios for this truss structure, the rate of strain for each member was obtained and illustrated in a single graphical representation. Figure 8 shows the member numbers while the Figure 9 shows the rate of strain variation for each member for individual loading scenarios. This rate of strain variation indicates the rate that a structural member experiences, but not the most impacted member. Seemingly, the rate of strain variation for members M1 and M2 becomes most accelerated when the combined load is applied, indicating that these members experience a sum rate of strain from the compression strain (i.e., compression is negative in this study). For M3, M4 and M13, the rate of strain is equalized, indicating that these members experience the lowest rate of strain under the combined loading scenario in comparison to the vertical and inclined loading scenarios when applied individually. Therefore, the combined loading scenario can be more efficient than the individual applied loadings (i.e., vertical and inclined). It can be noticed that when the lowest rate of strain is obtained, the member experiences the least strain impact. The digital twin development of this truss has the potential to obtain the rate of strain, and in some cases, the least effective member under different loading scenarios in real-time conditions. For M5, M8 and M10, the rate of strain is also equalized to some extent, where under vertical loading, the rate is in the high range of compression strain, and in inclined loading, the rate of strain is in the high range of tension strain. For the same members, the rate of strain is in the lower range of the tension strain when a combined load is applied. A similar observation was obtained for M9 while in the opposite strain mechanism. For members M6, M7 and M12, the rate of strain is at a zero value in all applied loading scenarios, indicating that these members are the least impacted members in the truss model. It can be determined that the placement of these members is due to the completion of the truss section, rather than the structural members. In this truss structure, M11 seems to have the highest rate of strain under the combined loading scenario and a low rate of strain under vertical loading, indicating that this member experiences an accelerated rate when vertical and inclined loadings are applied simultaneously. This observation obtained from the digital twin in real-time conditions can be significantly important for the evaluation of the members’ performance, typically for a large-scale structure.

### 4.2. Real-Time Visualization of Stress

**Figure 10:**
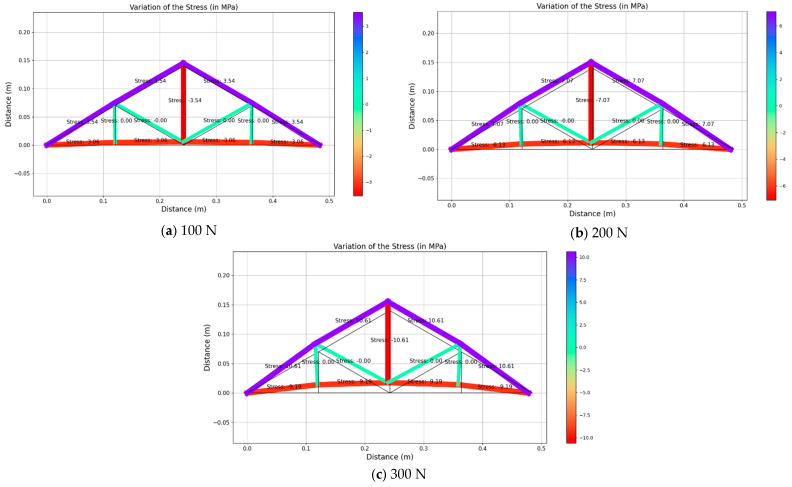
Variations of the structural response for vertical loading.

**Figure 11:**
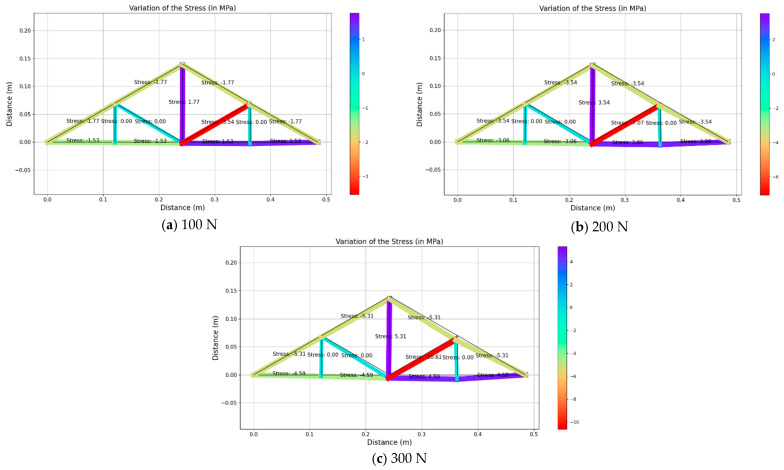
Variations of the structural response for inclined loading.

**Figure 12:**
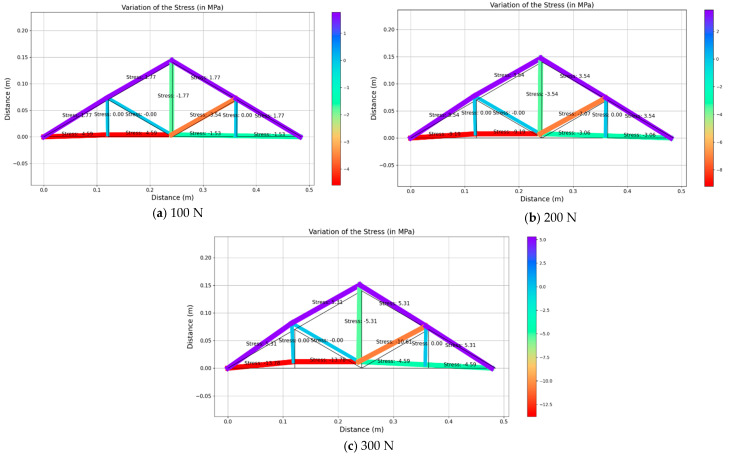
Variation of structural responses for combined loading.

Upon receiving the strain values from strain gauges in real-time, those values were used to back-calculate the applied load and thereby obtain the stress and deformed shape of the structure. As this structure behaves linearly, the stress of each member was obtained by using the linear relationship of Young’s modulus. However, the authors focused on deploying the inverse analysis of the truss model that was considered for the current study and the possibility of applying the same method for a larger structure. Figure 10, Figure 11 and Figure 12 illustrate the structural response for the three loading scenarios for three different magnitudes (i.e., 100 N, 200 N and 300 N).

It can be noticed that the un-deformed shape is shown in black and the deformed shape is in different colors, representing the ranges of stress values for each member. For instance, the red color indicates the highest compressive stress whilst the purple color indicates the highest tensile stress value.

When a vertical load is applied upwards at node C, all the members at the bottom of the truss show compressive stresses and all the members at the top of the truss show tensile stresses. The member that is in the middle of the truss also shows a compressive stress of 3.54 MPa, 7.07 MPa and 10.61 MPa for an applied loading of 100 N, 200 N and 300 N, respectively. Noticeably the same member always experiences the maximum compressive stress. Similarly, the members on the top side of the truss experience the maximum tensile stress values of 3.54 MPa, 7.07 MPa and 10.61 MPa for 100 N, 200 N and 300 N, respectively. Hence, those members are the most critical members experiencing real-time excessive stress. These output results clearly show the significance of the digital twin on the bridge structures. For the vertical loading scenario, the remaining members experience the minimum stresses (i.e., zero stresses). However, the existence of those members is important to maintain the shape formation and reduce the span of the member that is potentially experiencing high excessive compressive and tensile stresses. Additionally, it can be noticed that the stress values show a symmetrical distribution within the truss structure, which can be due to the symmetrical shape of the structure.

For an inclined load scenario, when a load is applied towards the truss structure at node H, member CH (Figure 3) experiences the maximum compressive stress values of 3.54 MPa, 7.07 MPa and 10.61 MPa for the loading magnitudes of 100 N, 200 N and 300 N, respectively, while member CG experiences the maximum tensile stress values of 1.77 MPa, 3.54 MPa and 5.31 MPa for the loading magnitudes of 100 N, 200 N and 300 N, respectively. Similar to the vertical loading scenario, higher deformation can be observed when increasing the load. Under the inclined loading scenario, some of the members experience similar stresses, while the stress distribution is not symmetrical for all of the members. It can be attributed to the unsymmetrical vector movement of the nodes leading to unsymmetrical loading on the members. Additionally, members BF, CF and DH experience the minimum stresses (i.e., zeros stress).

For the combination of the vertical load and inclined load applied on the structure, members AB, BC and CH (Figure 3) experienced higher compressive stresses, whilst members AB and BC experienced the highest compressive stress values of 4.59 MPa, 9.19 MPa and 13.78 MPa for the loading magnitudes of 100 N, 200 N and 300 N, respectively. Also, the members on the top side of the truss obtained the highest tensile stress value of 1.77 MPa, 3.54 MPa and 5.31 MPa for each incremented loading magnitude. Similar to the earlier loading scenarios, the structure deformed more when increasing the magnitude of the load. Overall, all these stress values under the three loading arrangements correlate with calculated stress values and thereby prove the high accuracy of this model. The effectiveness of the developed DT is significant when it is applied to the large-scale structure for the determination of the stress distribution when the structure experiences random applied loadings.

### 4.3. Real-Time Deflection/Displacement Variation

**Figure 13:**
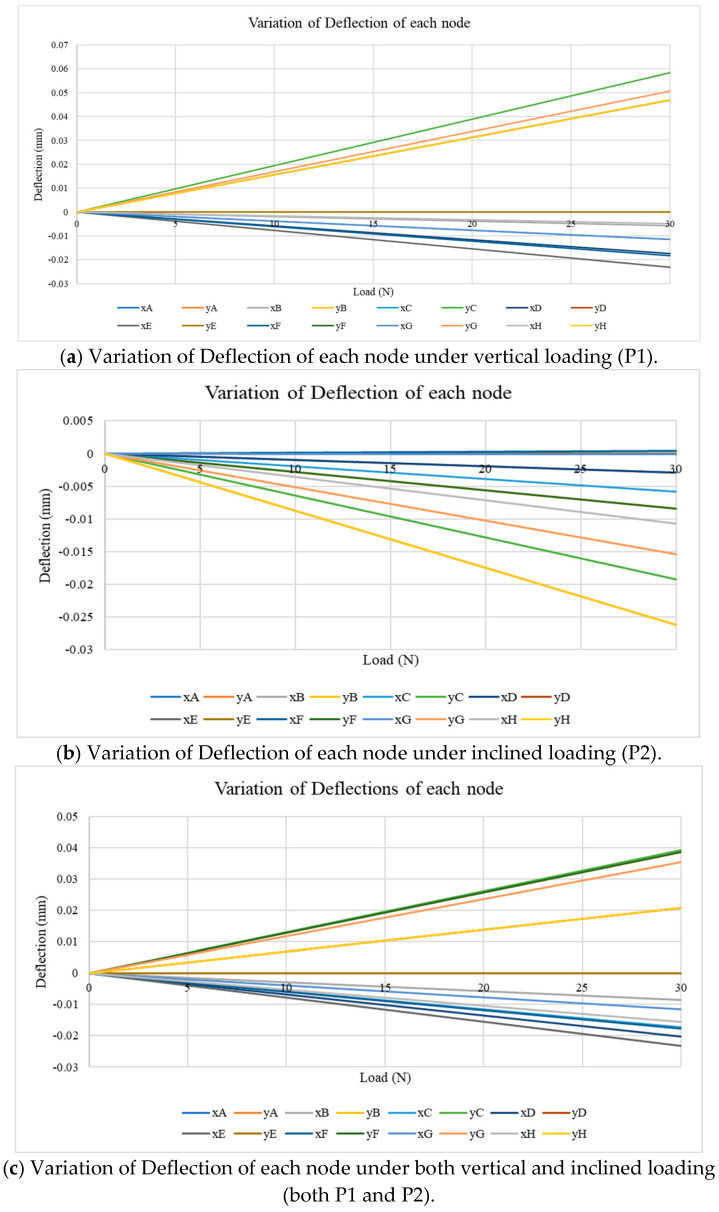
Variation of Deflection of each load scenario with time.

Further studies in relation to the deflection of the members and nodes have been evaluated by using the digital model. The deflection has been obtained for the horizontal and vertical direction, referred to as the x and y displacements, at each respective node (i.e., the letter refers to the name of the node). The applied load was increased from 0 to 300 N at a rate of 10 N/s for all loading scenarios (i.e., vertical, inclined and the combination of both) and the calculated deflection of each node from the digital model was monitored (Figure 13). Consequently, a linear variation of the deflection was observed at each node, except at the constrained nodes, which correlates to the expected structural response from a linearly elastic structure. Also, the constrained degrees of freedom of the nodes were zero throughout the entire period. From Figure 13a, when an upward vertical load is applied at node C, all the y-displacement values become positive, as expected in the initial stages of the experiment program, where the highest vertical deflection is at node C, which is approximately 5.8 × 10^−2^ mm for the 300 N load. Also, the maximum x-displacement can be observed in node E, which is approximately −2.3 × 10^−2^ mm for the 300 N load.

When the inclined load is applied at node H (Figure 13b), the positive variation has been obtained for the x-displacement of node F (i.e., towards the right side from the structure), and both node D and node H show the maximum y-displacement, which is approximately −2.6 × 10^−2^ mm at 300 N load. Also, node H shows the maximum x-displacement value, which is approximately 1.7 × 10^−2^ mm under 300 N. It can be noticed that from all of the nodes, node H is the most critical point where the deflection occurs for this particular loading scenario. This observation has a significant impact on the analytical application and visual observation obtained from the digital twin development, and these benefits are obtained when the digital twin is implemented in field applications. Alternatively, for the combined loading pattern, the highest y-displacement can be observed at node C, which is approximately 3.9 × 10^−2^ mm at 300 N, and the highest x-displacement can be observed at node E, which is approximately −2.3 × 10^−2^+ mm under 300 N as shown in Figure 13c. It has been noted that for both the vertical and combined loadings, the maximum y-displacement was observed at mid-span (node C), and this observation correlates with the classical beam theory. Also, the maximum vertical displacement remains small relative to the overall span (1/20,000), suggesting that the structure remains well within the elastic range and is stable under the tested loading conditions. Also, when comparing the x-displacements in all three scenarios, they were observed to be very minor, indicating no significant lateral sway or instability effects. Hence, it can be concluded that the boundary conditions and the symmetry of the truss model have provided an adequate restraint under the test loads. However, under real-world asymmetric conditions or under dynamic loading, lateral displacements and out-of-plane effects may become more pronounced and should be addressed in future 3D implementations.

**Figure 14:**
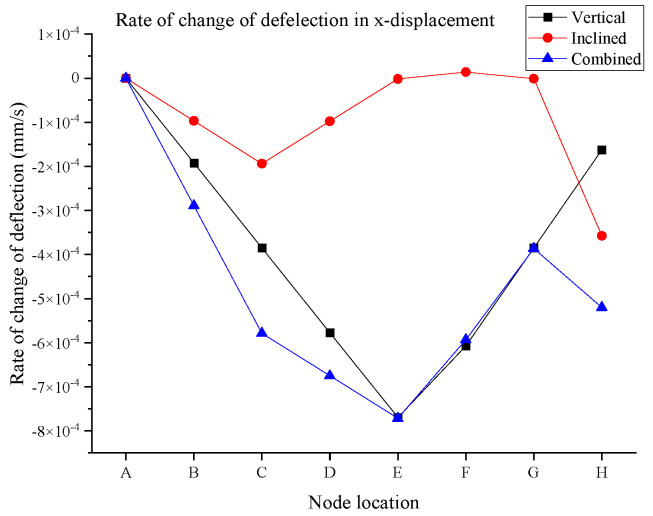
Rate of change in x-displacement.

**Figure 15:**
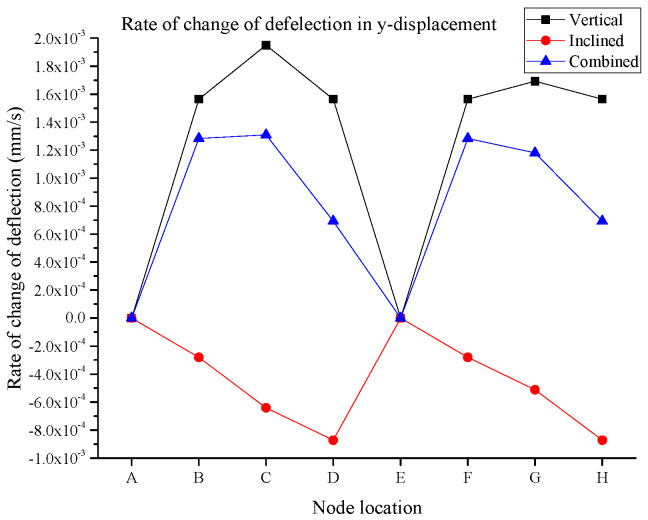
Rate of change in y-displacement.

Further to the deflection values obtained from the digital twin model, the rate of change in displacement is also evaluated for each node for the aforementioned three loading patterns. Figure 14 and Figure 15 show the rate of change in deflection for the x-displacement and y-displacement, respectively. Seemingly, the change in deflection for the vertical and combined scenarios is generally higher in comparison to the inclined loading scenario. In some cases, this rate is close to zero, indicating the nodes experience the last deflection. This indicates that the structure can behave very differently in lower impact conditions when inclined loading is applied to the structure. This is another significance of digital twin development, which demonstrates the deflection of the connection of the truss in real time for the field applications. Another indication is that the highest rate of change in x-displacement was observed for node 5 or node E and is approximately −7.69 × 10^−4^ mm/s for vertical loading, and it was slightly reduced to −7.15 × 10^−4^ mm/s due to the combined effect of both vertical and inclined loads.

Alternatively for the y-displacement, the highest rate of change in the deflection varies for the three different loading scenarios. Unlike the x-displacement, in the y-displacement, the rate of the deflection is generally equalizing amongst the three loading patterns for node E, where the rate deflection is close to zero, indicating that this node is experiencing the least vertical displacement when a combined load is applied. The significance of the digital development for this truss structure in real-time conditions demonstrates that when the structure behaves in drastic conditions such as vertical loading (i.e., upward), then we can have the smallest effect on structural behavior and can have some equalized node location by applying the inclined load simultaneously.

## 5. Limitations

This study demonstrated a physics-based digital twin framework for real-time structural response monitoring of a laboratory-scaled bridge. Even though the suggested methodology demonstrated promising results, several limitations are inherent in the current implementation. It is important to explore these limitations and to highlight promising avenues for future research within this domain.

1. This study successfully demonstrated a digital twin system of a 2D truss model to simulate the in-plane behavior of a typical truss bridge while capturing the dominant axial load paths. However, 3D spatial effects such as torsion, lateral bending or interactions with the deck of the truss bridge have not been accounted for. It can be suggested to consider a full 3D structural model to more accurately replicate real-world behavior for future work.
2. The experimental setup was conducted under controlled laboratory conditions, and hence, strain gauges were installed precisely, and boundary conditions were well defined. However, due to the environmental variability in real-world applications, it would be difficult to maintain such a controlled environment. Therefore, the stability and the accuracy of the inverse load identification method could be affected.
3. The current method demands accurate and dense strain gauge placement. Hence, the accuracy of this method may degrade due to missing or faulty sensors. Therefore, this system can be further upgraded to incorporate robustness strategies, such as noise filtering, regularization or adaptive sensor fusion.
4. The current method is entirely physics-based and does not yet incorporate a data-driven technique such as machine learning or any other statistical technique. The addition of such a system to the algorithm would improve the robustness of the current system and address the practical limitations of the current system when extending to large-scale systems through adaptive calibration and real-time error correction.

## 6. Conclusions

Structural health monitoring (SHM) system deployments are important for the safe operation of infrastructure. The development of the digital twin technique will be drastically beneficial for transportation networks. The digital twin has the potential to facilitate the real-time structural integrity assessment of an operating civil infrastructure (e.g., bridges). This study focused on the implementation of a novel technique to develop a digital twin for bridges, using the combination of the inverse load identification method and the finite element analysis method on a laboratory-scaled truss steel bridge. A Python script that is based on both the finite element method and inverse load identification method was developed, and the structural response was obtained in real-time from the strain gauges and then visualized.

The capacity of the digital twin system was analyzed under three main loading patterns that included a vertical load, an inclined load and a combination of them. The real-time visualizations included stress variations throughout the structure and the deflected shape. It was observed that the developed digital twin of the bridge responds to any variations in the actual structure accurately in near real-time conditions with a one-second spontaneous calculation. This enables the user to identify the location and the magnitude of any type of loading that is applied to the structure.

Furthermore, the developed model was highly responsive to the sudden changes of the applied load and to ensure the accuracy of the digital twin, the applied load was increased constantly and the responses from the strain and stress variations of each of the members of the truss and the variation of deflection were monitored instantly. Then, the values obtained were compared with the manually calculated values of each member and it was observed that those values were highly similar to each other. A linear variation in the strain and deflection was noticed when the applied load increased at a constant rate under the three loading patterns. The developed method can be extended to even a large-scale bridge with several adaptations. Field deployment would require alternative sensors that can withstand harsh environmental conditions, powerful data acquisition systems with wireless transmission capabilities and long-term power solutions, such as solar or battery modules. Additionally, for ensuring stability and synchronization in real-world conditions, the sensor readings would require periodic calibrations against reference sensors to mitigate the effects of sensor drift or communication delays due to extreme weather conditions. Also, it was observed that the present method requires strain readings in all of the elements of the truss bridge, and it will not function properly otherwise. It was further observed that this limitation can be avoided by combining a data-driven method for the same process, and this has been suggested as possible future work. Additionally, such a method will assist in accurately capturing the structural response of a real-world bridge even with sensor noise.

Compared to conventional SHM techniques, such as visual inspections, vibration-based monitoring, or model updating using modal parameters, the proposed method offers real-time insights into stress and deformation states throughout the structure. It not only detects the damage but also allows for more localized and proactive maintenance decision-making. However, several limitations exist in the current approach. It requires the presence of sensors in each member of the truss bridge to capture the structural response accurately, and it assumes linear elastic material behavior. Also, this method highly depends on the calibration and reliability of sensors, which can be challenging in real-world conditions. Accordingly, optimizing sensor configurations and integrating data-driven algorithms would significantly enhance the proposed methodology and are therefore recommended as key directions for future research.

## Data Availability

The data sets generated during the current study are available from the corresponding author on reasonable request.
